# The clinical and prognostic value of polo-like kinase 1 in lung squamous cell carcinoma patients: immunohistochemical analysis

**DOI:** 10.1042/BSR20170852

**Published:** 2017-08-14

**Authors:** Hefei Li, Haibo Wang, Zhenqing Sun, Qiang Guo, Hongyun Shi, Youchao Jia

**Affiliations:** 1 Department of Thoracic Surgery, Affiliated Hospital of Hebei University, Baoding 071000, Hebei Province, China; 2 Department of Radiation Oncology, Affiliated Hospital of Hebei University, Baoding 071000, Hebei Province, China; 3 Department of Oncology, Affiliated Hospital of Hebei University, Baoding 071000, Hebei Province, China

**Keywords:** biomarkers, immunohistochemistry, lung cancer, PLK1, pronosis

## Abstract

Polo-like kinase 1 (PLK1) has been suggested to serve as an oncogene in most human cancers. The aim of our study is to present more evidence about the clinical and prognostic value of PLK1 in lung squamous cell carcinoma patients. The status of PLK1 was observed in lung adenocarcinoma, lung squamous cell carcinoma, and normal lung tissues through analyzing microarray dataset (GEO accession numbers: GSE1213 and GSE 3627). *PLK1* mRNA and protein expressions were detected in lung squamous cell carcinoma and normal lung tissues by using quantitative real-time PCR (qRT-PCR) and immunohistochemistry. In our results, the levels of PLK1 in lung squamous cell carcinoma tissues were higher than that in lung adenocarcinoma tissues. Compared with paired adjacent normal lung tissues, the PLK1 expression was increased in lung squamous cell carcinoma tissues. Furthermore, high expression of PLK1 protein was correlated with differentiated degree, clinical stage, tumor size, lymph node metastasis, and distant metastasis. The univariate and multivariate analyses showed PLK1 protein high expression was an unfavorable prognostic biomarker for lung squamous cell carcinoma patients. In conclusion, high expression of PLK1 is associated with the aggressive progression and poor prognosis in lung squamous cell carcinoma patients.

## Introduction

The incidence and mortality of lung cancer are the highest amongst malignant tumors worldwide [1]. Based on the 2015 Cancer Statistics in China, lung cancer alone is expected to account for 17% of all new cancer cases, and a total of estimated 4291600 new cancer cases occurred in 2015 [2]. In the United States, lung cancer also remains to be the leading cause of cancer deaths with a total of estimated 155870 lung cancer deaths in 2017 according to 2017 American Cancer Statistics [3]. Lung squamous cell carcinoma and lung adenocarcinoma are the main histological subtypes of lung cancer [4]. In recent years, the survival of lung adenocarcinoma patients have had a great progress due to the development of molecular target therapy (such as EGFR-TKI and ALK inhibition) [5,6]. However, lung squamous cell carcinoma still lacks effective molecular target for developing target therapy. Therefore, it is necessary to explore novel biomarkers for prognosis prediction and development of molecular target therapy for lung squamous cell carcinoma patients.

Polo-like kinase 1 (PLK1) is a member of the mitotic serine/threonine kinases family, which were originally identified in the fruitfly, *Drosophila melanogaster* from mutants with abnormal spindle poles [7]. PLK1 has been found to be overexpressed in many types of malignant human tumors and facilitate tumor cell proliferation [8]. In lung adenocarcinoma, down-regulation of PLK1 expression obviously suppressed cell proliferation and induced cell cycle arrest and apoptosis *in vitro* [9–11], and the combination of PLK1-shRNA and low-dose gemcitabine produced an additive antitumor activity on the lung tumors *in vivo* [12]. In non-small cell lung cancer, silenced PLK1 expression by iNOP-7-PLK1 siRNA reduced tumor growth *in vitro* and *in vivo* [13], and PLK1 was the target for *miR-100* to regulate non-small cell lung cancer cell proliferation, apoptosis, and cell cycle [14]. The clinical study in non-small cell lung cancer patients showed that PLK1’s high expression was markedly associated with advanced clinical stage, higher tumor classification, and lymph node metastasis, and was an independent unfavorable prognostic biomarker for non-small cell lung cancer patients [15]. In our initial study, we analyzed microarray datasets, and found PLK1 overexpressed in lung squamous cell carcinoma tissues compared with lung adenocarcinoma tissues. We further confirmed the status of *PLK1* mRNA and protein expressions in lung squamous cell carcinoma tissue samples. Thus, we supposed that PLK1 may serve as an important biomarker for lung squamous cell carcinoma patients. The aim of our study was to explore the clinical and prognostic significance of PLK1 in lung squamous cell carcinoma patients.

## Materials and methods

### Analysis of microarray data

Microarray dataset (GEO accession number: GSE1213) from five pairs of squamous lung cancer specimens and adjacent normal lung specimens was submitted by Wachi et al. [16]. Microarray dataset (GEO accession number: GSE 3627), which was composed of 42 lung adenocarcinoma specimens and 18 lung squamous cell carcinoma specimens, was submitted by Kuner et al. [17]. The differentially expressed genes were screened and identified by quantitative real-time PCR (qRT-PCR) for the following study.

### Patients and specimens

The study was approved by the Ethical Committees of Affiliated Hospital of Hebei University. An informed consent was obtained from all the participants before enrollment in the study. The entire study was performed based on the Declaration of Helsinki. A total of 132 lung squamous cell carcinoma tissues and 33 adjacent normal lung tissues were collected at Affiliated Hospital of Hebei University between January 2008 and December 2015. Tissues were respectively stored in liquid nitrogen for qRT-PCR and formaldehyde solution for immunohistochemistry. None of the patients in the present study had received neoadjuvant antitumor treatment. Clinical staging and system treatment were based on the seventh edition of the American Joint Committee on Cancer (AJCC) Cancer Staging Manual and National Comprehensive Cancer Network (NCCN) guidelines, respectively.

### qRT-PCR

Total RNA was extracted from tissues’ cell lines using RNAiso Plus (Takara), and then reverse-transcribed to cDNAs using the PrimeScript RT Master Mix (Takara), according to manufacturer’s instructions. The LightCycler (Roche, U.S.A.) was selected to conduct the amplification of cDNAs using SYBR Premix Ex Taq™ II (Takara). The primers used for RT-PCR were purchased from Takara. Primers for the human PLK: forward primer, 5′-GATTCCACGGCTTTTTCGAG-3′; reverse primer, 5′-CCCACACAGGGTCTTCTTCC-3′. Primers for the human GAPDH: forward primer, 5′-ACCACAGTCCATGCCATCAC-3′; reverse primer, 5′-TCCACCACCCTGTTGCTGTA-3′. Relative expression was calculated via the comparative cycle threshold method and was normalized to the expression of GAPDH.

### Immunohistochemistry

Immunohistochemical analysis was performed to measure PLK1 protein expression in 132 lung squamous cell carcinoma tissues and 33 adjacent normal lung tissues. In brief, slides were baked at 60°C for 1 h, followed by deparaffinization with xylene, and rehydrated. The sections were submerged in EDTA antigenic retrieval buffer and microwaved for antigen retrieval. They were then treated with 3% hydrogen peroxide in methanol to quench endogenous peroxidase activity, followed by incubation with 5% BSA to block nonspecific binding. Sections were incubated with anti-PLK1 (1:150 dilution, Abcam) overnight at 4°C. After washing, tissue sections were treated with secondary antibody, followed by incubation with conjugated horseradish peroxidase streptavidin. Tissue sections were then counterstained with Hematoxylin, dehydrated, and mounted. Finally, sections were viewed under a bright-field microscope.

### Evaluation of staining

The tissue sections stained immunohistochemically for PLK1 were reviewed and scored separately by two pathologists blinded to the clinical parameters. Any disagreements were arbitrated by the third pathologist. For PLK1 assessment, staining intensity was scored as 0: negative; 1: weak; 2: moderate; or 3: strong, and staining extent was scored as 0, 0%; 1, 1–10%; 2, 11–50%; 3, 51–80%; or 4, more than 80% positive cells. The final score was calculated by multiplication of these two variables. Low expression of PLK1 was defined as 0–6 score; high expression of PLK1 was defined as more than 6 score.

### Statistical analysis

Diagrams were conducted using GraphPad Prism 5.0 and statistical analyses were accomplished using SPSS 17.0. The association between clinicopathological characteristics and PLK1 expression was determined using χ^2^ tests. Survival analysis was performed using Kaplan–Meier method. Univariate and multivariate Cox regression models were used to evaluate prognostic significance. The comparison between the two groups was conducted using *t* test. *P*-values in all experiments were considered statistically significant at less than or equal to 0.05.

### Ethics statement

The present study was approved by the Research Ethics Committee of Affiliated Hospital of Hebei University. The informed written consents were collected from all eligible patients and the entire study was performed based on the Declaration of Helsinki.

## Results

### PLK1 is overexpressed in lung squamous cell carcinoma

**Figure 1:**
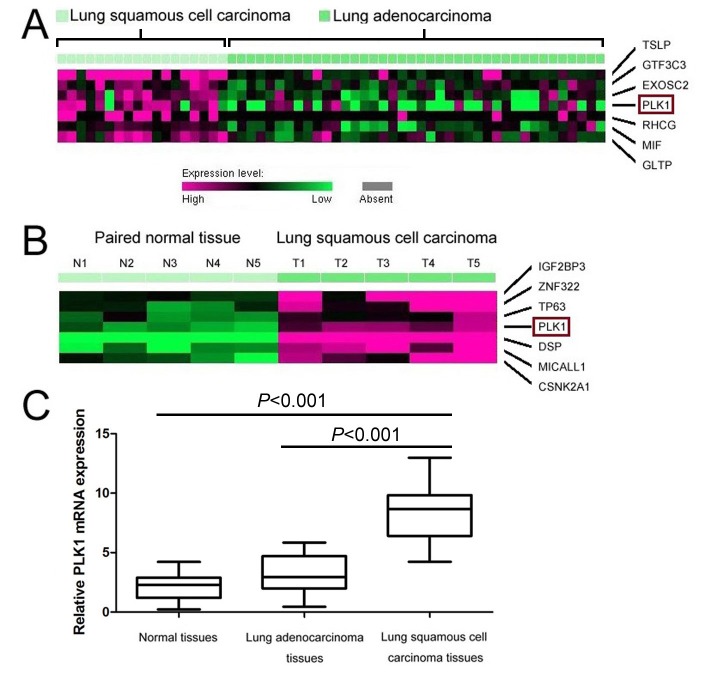
The status of PLK1 in lung squamous cell carcinoma, lung adenocarcinoma, and normal lung tissues (**A**) PLK1’s high expression was observed in lung squamous cell carcinoma tissues compared with lung adenocarcinoma tissues by microarray data (GSE3627). (**B**) Expression of PLK1 was increased in lung squamous cell carcinoma tissues compared with adjacent normal lung tissues through observation of microarray data (GSE1213). (**C**) *PLK1* mRNA expression was detected in lung squamous cell carcinoma, lung adenocarcinoma, and normal lung tissues by qRT-PCR.

In order to explore novel biomarkers for lung squamous cell carcinoma patients, microarray datasets (GSE1213 and GSE 3627) were analyzed. Based on Kuner et al. [17] microarray data (GSE3267), PLK1 was found overexpressed in lung squamous cell carcinoma tissues compared with lung adenocarcinoma tissues (Figure 1A). Furthermore, we verified the status of PLK1 between lung squamous cell carcinoma and lung adenocarcinoma through qRT-PCR, and found that the levels of *PLK1* mRNA was overexpressed in lung squamous cell carcinoma tissues compared with lung adenocarcinoma tissues with an average increase of 2.65-fold (*P*<0.001, Figure 1C).

**Figure 2:**
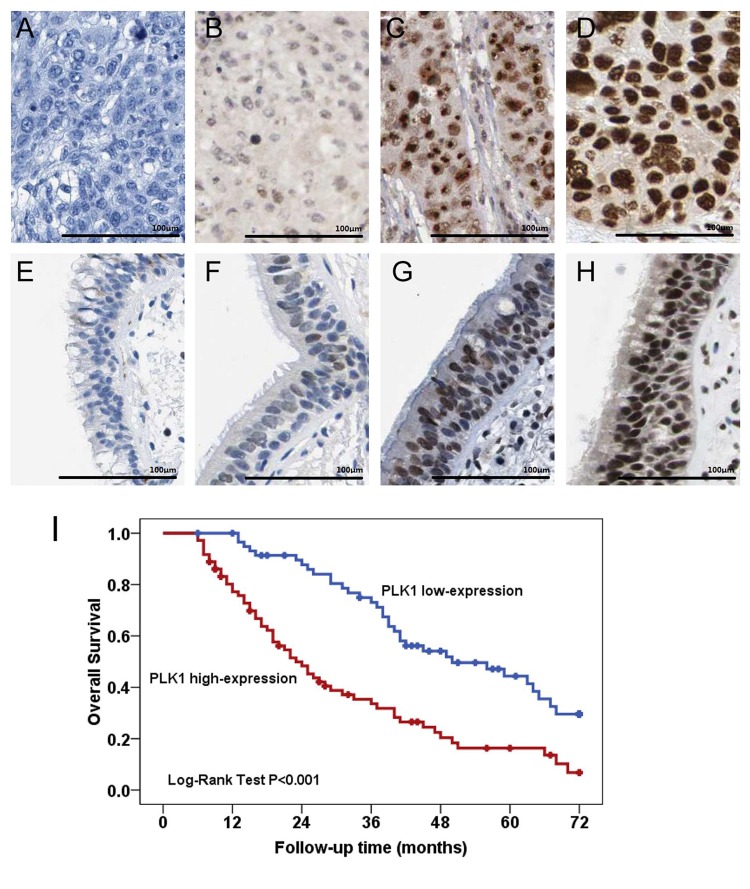
Immunohistochemical staining and pronostic value of PLK1 in lung squamous cell carcinoma (**A**–**H**) Immunohistochemical staining of PLK1 in lung squamous cell carcinoma tissues and adjacent normal tissues. (A) Negative expression of PLK1 in lung squamous cell carcinoma tissue. (B) Weak expression of PLK11 in lung squamous cell carcinoma tissue. (C) Moderate expression of PLK1 in lung squamous cell carcinoma tissue. (D) Strong expression of PLK1 in lung squamous cell carcinoma tissue. (E) Negative expression of PLK1 in normal bronchial epithelial tissues. (F) Weak expression of PLK1 in normal bronchial epithelial tissues. (G) Moderate expression of PLK1 in normal bronchial epithelial tissues. (H) Strong expression of PLK1 in normal bronchial epithelial tissues. (**I**) PLK1 protein overexpression is an unfavorable prognostic factor in lung squamous cell carcinoma patients. Kaplan–Meier survival analysis of overall survival duration in 132 lung squamous cell carcinoma patients according to PLK1 protein expression.

Moreover, we observed that the expression of PLK1 was increased in lung squamous cell carcinoma tissues compared with paired adjacent normal lung tissues from Wachi et al. [16] microarray data (GSE1213) (Figure 1B). Compared with paired adjacent normal lung tissues, the levels of *PLK1* mRNA was overexpressed in lung squamous cell carcinoma tissues with an average increase of 3.85-fold (*P*<0.001, Figure 1C). Furthermore, PLK1 protein expressions in 132 lung squamous cell carcinoma tissues and 33 adjacent normal lung tissues were detected by immunohistochemical staining (Figure 2A–H). We observed that PLK1 protein expression was increased in lung squamous cell carcinoma tissues in 54.5% (72/132) compared with adjacent normal tissues in 21.2% (7/33) (*P*=0.001, Table 1).

**Table 1:** Expression of PLK1 protein between lung squamous cell carcinoma and normal bronchial epithelial tissues

Group	Cases	PLK1		*P*-value
		High expression	Low expression	
Tumor tissues	132	72	60	0.001
Normal tissues	33	7	26	

### PLK1 protein expression is associated with malignant status of lung squamous cell carcinoma patients

The clinical significance of PLK1 protein expression in lung squamous cell carcinoma patients was explored in 132 lung squamous cell carcinoma tissue specimens through immunohistochemistry. The association between clinicopathological features and PLK1 protein expression in lung squamous cell carcinoma patients was summarized in Table 2. We observed that PLK1 protein’s high expression positively correlated with differentiated degree (high or middle compared with low, *P*<0.001), clinical stage (I–II compared with III–IV, *P*<0.001), tumor size (T1–T2 compared with T3–T4, *P*<0.001), lymph node metastasis (N1 compared with N2–N3, *P*<0.001), and distant metastasis (M0 compared with M1, *P*=0.002). However, there were no significant relationships between PLK1 protein expression and age (*P*=0.592), gender (*P*=0.746), smoking (*P*=0.108), and tumor size (*P*=0.105).

**Table 2:** Associations between clinicopathologic factors and PLK1 in lung squamous cell carcinoma patients

Characteristics	*n*	PLK1		*P*-value
		High expression	Low expression	
Gender				
Female	45	26	19	0.592
Male	87	46	41	
Age (y)				
<50	53	28	25	0.746
≥50	79	44	35	
Smoking				
No	76	46	30	0.108
Yes	56	26	30	
Differentiated degree				
High or middle	79	32	47	<0.001
Low	53	40	13	
Clinical stage				
I–II	54	14	40	<0.001
III–IV	78	58	20	
Tumor size				
T1–T2	69	33	36	0.105
T3–T4	63	39	24	
Lymph node metastasis				
N0–N1	62	20	42	<0.001
N2–N3	70	52	18	
Distant metastasis				
M0	121	61	60	0.002
M1	11	11	0	

### PLK1 protein overexpression is an unfavorable prognostic factor in lung squamous cell carcinoma patients

The prognostic value of PLK1 protein expression in lung squamous cell carcinoma patients was also explored in 132 lung squamous cell carcinoma tissue specimens through immunohistochemistry. Kaplan–Meier survival analysis showed lung squamous cell carcinoma patients, that expressed high level of PLK1 protein, had lower overall survival compared with patients with low level of PLK1 protein expression (*P*<0.001, Figure 2I). Moreover, we conducted univariate analysis and identified five prognostic parameters: clinical stage, tumor size, lymph node metastasis, distant metastasis, and PLK1 protein expression. Finally, multivariate analysis showed PLK1 protein’s high expression was an independent poor prognostic factor for lung squamous cell carcinoma patients (hazard ratio (HR) =1.926, 95% CI: 1.199–3.093, *P*=0.007, Table 3).

**Table 3:** Univariate and multivariate analyses of overall survival in lung squamous cell carcinoma patients

Parameters	Univariate analysis			Multivariate analysis		
	*P*-value	HR	95% CI	*P*-value	HR	95% CI
Gender						
(Female compared with male)	0.471	1.185	0.747–1.882			
Age						
(<50 compared with ≥50years)	0.149	0.737	0.487–1.116			
Smoking						
(No compared with yes)	0.653	1.102	0.723–1.680			
Differentiated degree						
(High or middle compared with low)	0.869	0.965	0.629–1.479			
Clinical stage						
(I–II compared with III–IV)	<0.001	3.214	2.009–5.141	0.448	0.615	0.175–2.160
Tumor size						
(T1–T2 compared with T3–T4)	0.006	1.809	1.189–2.752	0.076	1.517	0.958–2.404
Lymph node metastasis						
(N0–N1 compared with N2–N3)	<0.001	3.614	2.285–5.716	0.024	3.997	1.197–13.347
Distant metastasis						
(M0 compared with M1)	<0.001	6.037	3.030–12.030	0.013	2.541	1.218–5.302
PLK1 expression						
(Low compared with high)	<0.001	2.591	1.682–3.991	0.007	1.926	1.199–3.093

## Discussion

PLK1 is a member of the family of mitotic serine/threonine kinases, which are characterized by a C-terminal polo-box domain and a conserved N-terminal catalytic domain [18,19]. PLK1 homologs were originally identified as Polo in *Drosophila*, Plo1 in *Schizosaccharomyces pombe*, Cdc5 in *Saccharomyces cerevisiae*, and Plx1 in *Xenopus* [20,21]. PLK1 plays a key role in mitotic entry of proliferating cells and regulates many aspects of mitosis [22]. PLK1 protein levels are lowest during G_1_, elevate S, and enrich their greatest concentration in G_2_/M [23].

Recently, PLK1 has been found to be overexpressed in many types of human cancers such as hepatocellular carcinoma [24–28], glioma [29–32], colorectal cancer [33–38], gastric cancer [39–42], pancreatic cancer [43–45], lymphomas [46,47], ovarian cancer [48,49], bladder cancer [50], prostate cancer [51], breast cancer [52,53], and esophageal cancer [54]. In non-small- cell lung cancer, PLK1 has been shown to be overexpressed in tumor tissues and cell lines [15,55]. Similarly, our study also showed that PLK1 expression was increased in lung cancer tissues. Interestingly, we further found the levels of PLK1 in lung squamous cell carcinoma tissues were higher than that in lung adenocarcinoma tissues. The difference of PLK1 between lung squamous cell cancer and lung adenocarcinoma would be most likely to be due to tumor heterogenicity. Thus, we sequentially explored that clinical significance of PLK1 in lung squamous cell carcinoma patients, and found high expression of PLK1 protein was correlated with differentiated degree, clinical stage, tumor size, lymph node metastasis, and distant metastasis. Analogous to a report from Wang et al. [15], their research showed that PLK1 overexpression was markedly associated with advanced clinical stage, higher tumor classification, and lymph node metastasis in non-small cell lung cancer patients [15]. In colorectal cancers, Takahashi et al. [33] demonstrated that elevated expression of PLK1 was associated with pT (primary tumor invasion), pN (regional lymph nodes), and the Dukes’ classification. Moreover, Weichert et al. [56] reported that PLK1 expression was correlated with tumor grade, vascular invasion, HER2 expression, and markers of proliferative activity in breast cancer patients. In hepatocellular carcinoma patients, He et al. [26] suggested that the PLK1 positive expression was correlated with venous invasion tumor nodules and Edmondson grade. Liu et al. [57] found that PLK1 expression had associations with systemic symptom (fever, night sweats, and weight loss), lactate dehydrogenase (LDH) level, and International Prognostic Index (IPI) scores. These studies consistently suggested that PLK1 expression was positively associated with malignant status of human cancers.

In recent decades, PLK1 overexpression has been suggested to serve as a prognostic factor in most types of human cancers. Linton et al. [58] showed that PLK1 expression in over 10% of tumor cells is associated markedly with an unfavorable prognosis in malignant pleural mesothelioma patients. Moreover, Yamada et al. [24] reported hepatoblastoma patients with high expression of PLK1 represented obviously poorer outcomes than those with PLK1 low expression. In children with medulloblastoma, PLK1 was independently correlated with poor outcomes through using Cox regression analyses in two patient cohorts [59]. In ovarian cancer patients, Weichert et al. [48] found that PLK1 overexpression was an independent unfavorable prognostic marker through univariate and multivariate survival analyses. Doniz et al. [60] suggested high expression of PLK1 was correlated with shorter cancer-specific overall survival and disease-free survival in breast cancer patients. In non-small cell lung cancer patients, Wang et al. [15] revealed that high PLK1 protein expression was an independent prognostic biomarker. In the present study, we further presented the evidence that lung squamous cell carcinoma patients that expressed high level of PLK1 protein had lower overall survival compared with patients with low level of PLK1 protein expression, and PLK1 protein high expression was an independent poor prognostic factor based on univariate and multivariate survival analyses.

Recently, PLK1 inhibitors have been considered to be anticancer drug candidates [8]. PLK1 inhibitors include BI 2536 (Boehringer Ingelheim Pharma, Germany) [61–63], Volasertib (BI 6727; Boehringer Ingelheim Pharma, Germany) [64], GSK4661364A (GlaxoSmithKline, U.K.) [65], HMN-214 (Nippon Shinyaku Co. Ltd, Japan) [66], NMS-P937 (Nerviano Medical Science, Italy) [67], TAK-960 (Tekmira Pharmaceuticals Co., Canada) [68], and Rigosertib (Onconova Therapeutics Inc., U.S.A.) [69]. Nowadays, PLK1 inhibitors are being used in several types of cancer patients to monitor their efficacy and safety in clinical trials. BI 2536 was the first selective PLK1 inhibitor investigated in clinical trials with patients who have various advanced and/or metastatic solid tumors including non-small cell lung cancer, hepatocellular carcinoma, colorectal cancer, melanoma, and ovarian cancer [61]. This first-in-man, phase I, dose-escalation study showed that BI 2536 was favorable in terms of manageable toxicity, high distribution into tissue, and favorable efficacy in cancer patients, which initiated further clinical studies [61]. The phase II trial with BI 2536 was carried out in 95 stage IIIB/IV non-small cell lung cancer to have modest clinical efficacy [62]. This report suggested modest efficacy and acceptable safety of BI 2536 monotherapy in relapse non-small cell lung cancer patients. Unfortunately, BI 2536 had a relatively poor clinical efficacy, with 4.2% patients achieving a partial response [62]. Thus, further clinical studies were mainly carried out in combination with other anticancer drugs rather than monotherapy of BI 2536. The combination therapy with the standard-dose pemetrexed was performed in 41 patients with non-small cell lung cancer [63]. This open-label, phase I study showed that BI 2536 (200 mg) combined with standard-dose pemetrexed has an acceptable safety profile, and had a relatively favorable clinical efficacy, with 95.1% patients evaluating tumor response [63]. In our study, we found that the levels of PLK1 were higher in in lung squamous cell carcinoma tissues compared with lung adenocarcinoma tissues, and PLK1 high expression was the aggressive progression and poor prognosis in lung squamous cell carcinoma patients, which implied that PLK inhibitors may have surprising clinical efficacy in lung squamous cell carcinoma patients. Further studies would be needed to verify this supposition and evaluate the clinical efficacy of PLK inhibitors in lung squamous cell carcinoma patients.

In conclusion, PLK1 expression is increased in lung squamous cell carcinoma tissues, and associated with malignant status and prognosis in lung squamous cell carcinoma patients. PLK1 is an independent unfavorable prognostic factor for lung squamous cell carcinoma patients.

## Abbreviations

HR: hazard ratio
PLK1: polo-like kinase 1
95% CI: 95% confidence interval
